# Differences of macrophages in the tumor microenvironment as an underlying key factor in glioma patients

**DOI:** 10.3389/fimmu.2022.1028937

**Published:** 2022-10-31

**Authors:** Yangyang Wang, Yan Liu, Chengkai Zhang, Chuanbao Zhang, Xiudong Guan, Wang Jia

**Affiliations:** ^1^ Department of Neurosurgery, Beijing Tiantan Hospital, Capital Medical University, Beijing, China; ^2^ Beijing Neurosurgical Institute, Capital Medical University, Beijing, China; ^3^ China National Clinical Research Center for Neurological Diseases (NCRC-ND), Beijing, China

**Keywords:** glioma, tumor microenvironment, macrophage, risk model, multi-omics

## Abstract

**Background:**

Macrophages, the major immune cells in glioma microenvironment, are closely related to tumor prognosis. Further studies are needed to investigate macrophages, which will be helpful to fully understand the role of it and early achieve clinical translation.

**Methods:**

A total of 1334 glioma cases were enrolled in this study from 3 databases. In our works, the single cell cohorts from GSE89567, GSE84465, and the Chinese Glioma Genome Atlas (CGGA) datasets were used to analyze the key genes of macrophage. The bulk sequencing data from the Cancer Genome Atlas (TCGA) and CGGA datasets were respectively divided into the training set and validation set to test prognostic value of the key genes from single cell analysis.

**Results:**

Quantitative and functional differences significantly emerge in macrophage clusters between LGG and GBM. Firstly, we used the Seurat R package to identify 281 genes differentially expressed genes in macrophage clusters between LGG and GBM. Furthermore, based on these genes, we developed a predictive risk model to predict prognosis and reflect the immune microenvironment in glioma. The risk score calculation formula was yielded as follows: Risk score = (0.11 × EXP_MACC1_) + (−0.31 × EXP_OTUD1_) + (−0.09 × EXP_TCHH_) + (0.26 × EXP_ADPRH_) + (-0.40× EXP_ABCG2_) + (0.21 × EXP_PLBD1_) + (0.12 × EXP_ANG_) + (0.29 × EXP_QPCT_). The risk score was independently related to prognosis. Further, significant differences existed in immunological characteristics between the low- and high-risk score groups. What is more, mutation analysis found different genomic patterns associated with the risk score.

**Conclusion:**

This study further confirms that the proportion of macrophage infiltration is not only significantly different, but the function of them is also different. The signature, identified from the differentially expressed macrophage-related genes impacts poor prognosis and short overall survival and may act as therapeutic targets in the future.

## Introduction

Glioma is the most prominent malignancy of the central nervous system (CNS) in adults, and it is associated with an elevated recurrence rate, morbidity, and mortality (1). Despite comprehensive intervention involving surgical resection, radio-, and chemotherapies, patients often experience very poor outcome (2). Recently, a myriad of biological indicators were identified to facilitate the accurate diagnosis and prognosis of multiple cancer patients. In 2016, the World Health Organization (WHO) made revisions to their stratification of CNS tumors, based on morphology and molecular variables, thus, indicating that molecular evaluation is crucial to glioma diagnosis (3). However, despite much improvement in the molecular profile-based diagnosis and prognosis, patient outcomes are still unsatisfactory, so it requires further enhancement (4). As a result, it is critical to develop additional and better fit molecular models.

The tumor microenvironment (TME) mainly refers to the microenvironment associated with immune cells (5). TME is a vital component of tumor biology. Multiple reports suggested that associations between TME components like tumor cells or tumor-infiltrating immune cells strongly influence patient outcomes (6). Hence, TME is increasingly studied in the tumor research field. Tumor-associated macrophages (TAMs) are the primary invading immune cells within the glioma TME, and it accounts for 30~50% of all cells in TME (7). This raises the possibility that targeting TAMs may emerge as an attractive adjuvant therapy for glioma. In the past decade, high-throughput technologies produced a massive amount of biological data, including single-cell RNA sequencing data (scRNA-seq), transcriptomic sequencing data, genomic sequencing data, and so on. In addition, further mining and analyses of these data contributed to the exploration of valuable markers that can guide clinical treatment. Herein, we examined macrophages within the glioma TME, based on the multi-omics data, and revealed that both the proportion and function of macrophages differed between the lower grade glioma (LGG) and glioblastoma (GBM).

## Methods and materials

### Patients and datasets

The scRNA-seq data of 26 glioma cases, including 13 LGG and 13 GBM cases, were downloaded from the GEO and CGGA databases (8–10). A total of 16078 single cells were obtained in this study. Bulk sequencing data, as well as matched clinical patient profiles, of glioma patients were acquired from the TCGA cohort, CGGA cohort1, and CGGA cohort2, which included 623, 412, and 273 cases, respectively. The detail information was supplemented in **Table S1**. Subsequently, the RNA sequencing data were normalized. In a dataset that had several rows for the same gene, the values from all rows were averaged by the limma package, prior to computation *via* RPKM (reads per kilobase transcriptome per million reads) (11). Overall survival (OS) was described as the period between diagnosis date and date of last follow-up or death. This investigation received ethical approval from the Beijing Tiantan Hospital, an affiliation of the Capital Medical University.

### Processing and analysis of the glioma scRNA-seq data

**Figure 1:**
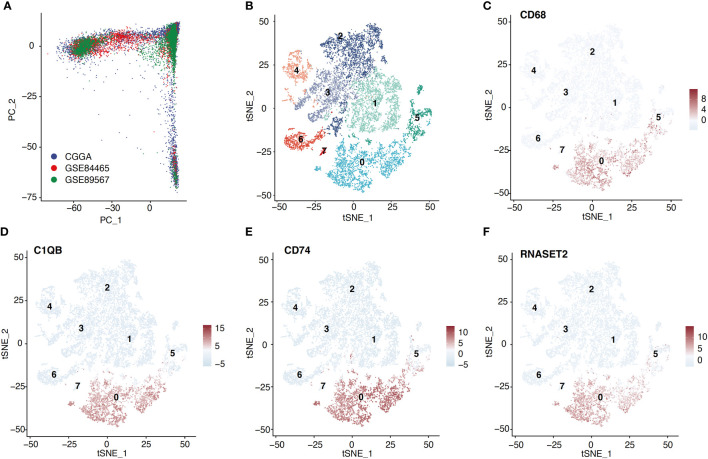
Macrophage cluster was identified based on scRNA-seq data. **(A)** ScRNA-seq data from three cohorts were shown based on the PCA algorithm. **(B)** The tSNE algorithm was applied for dimensionality reduction and 8 cell clusters were successfully classified. **(C–F)** tSNE plots show the marker genes expression for macrophage.

The Seurat R package was employed for scRNA-seq data analysis (12). Quality control was achieved by excluding low-quality genes present in < 3 cells, or low-quality cells containing < 100 total identified genes, or cells containing > 10% mitochondrial genes. Subsequently, the remaining data was normalized using the SCTransform method, thus properly eliminating the batch effects (**Figure 1A**). Principal component analysis (PCA) was employed to reduce scRNA-seq data dimension (13). In short, 30 principal components were employed for T-distributed stochastic neighbor embedding (tSNE). Then, the macrophage cluster was annotated and identified based on the CellMarker database (14).

### Differential and enrichment analysis

First, with the min-pct set at 0.3, log2 fold change > 3, and p.adj < 0.05, the macrophage related marker genes (MRGs) were computed using the Seurat function FindAllMarkers (12). By this approach, the marker genes, most highly expressed in macrophage, were identified, and they were described as macrophage related genes (MRG). Second, we compared the differentially expressed genes (DEGs) of the macrophage cluster between the LGG and GBM, using the Seurat function FindMarkers (filter: log2|fold change| > 1, p.adj < 0.05). Third, we selected the intersection between the two aforementioned sets, and defined them as differentially expressed macrophage-related genes (DE-MRGs), which differentially expressed in the macrophages from LGG compared with macrophages from GBM. Subsequently, we conducted enrichment analyses, using GO and KEGG tools, on the DE-MRGs *via* the R package clusterProfiler (15). P < 0.05 was deemed significant.

### Building the predictive model using COX regression and LASSO analysis

We further evaluated whether DE-MRGs were associated with OS, based on the bulk sequencing data. Using univariate Cox analysis *via* the “survival” R package, least absolute shrinkage and selector operation (LASSO) algorithm *via* the “glmnet” R package, and multivariable Cox regression, we identified 8 genes and corresponding coefficients. The risk scores (RS) were computed as follows: sum [coefficient(gene_i_) × expr(gene_i_)].

### The immunological role of RS in glioma TME

Immunological characteristics of the TME in glioma were evaluated in five ways. It included the immunomodulators (IM) expression, the expression of inhibitory immune checkpoints (IIC) and tumor-infiltrating immune cells (TIIC) effector genes, the cancer immunity cycle (CIC), the infiltration level of TIICs, and the function of macrophage. To do this, we first obtained 122 IMs, based on a prior investigation (16), which included MHC, receptors, chemokines, and immunostimulators. Second, we also obtained 18 IICs (17), as well as some TIICs effector genes from the Hu J study (18). Third, as reported in a prior investigation (19), the CIC reflects the anticancer immune response and comprises seven steps: release of cancer cell antigens (Step 1), cancer antigen presentation (Step 2), priming and activation (Step 3), trafficking of immune cells to tumors (Step 4), infiltration of immune cells into tumors (Step 5), recognition of cancer cells by T cells (Step 6), and killing of cancer cells (Step 7). The activities of these steps demonstrate the status of anti-cancer immunity, which was calculated by the website tool (19). Fourth, exploration of the proportion of TIICs is one of the most important parts of the assessment of TME. Following this, to avoid any error or bias by using a single algorithm, we comprehensively inferred the infiltration level of TIICs using seven independent algorithms: QuanTIseq, XCELL, and EPIC (20) which can play the role of mutual verification. Fifth, we further explored the relation between RS and the function of macrophage. One of the most important functions of macrophage is related to inflammatory cytokines (21, 22). We explored the relationship between RS and classical chemokines and surface markers of both M1-macrophages (IL12A, IL-12B, IL-23A, IL-23R, TNF) and M2-macrophages (IL-10, IL-4, IL-13, TGF-beta 1, TGF-beta 2, TGF-beta 3). Subsequently, we further explored the correlation of RS and the five aforementioned variables in three cohorts.

### Enrichment analysis of RS in glioma

Correlation analysis was conducted between RS and gene expression. After that, enrichment analysis, including GO analysis and KEGG analysis, was applied for the correlated genes (|r| > 0.5, P < 0.05). We conducted enrichment analyses on the correlated genes *via* the R package clusterProfile (15). P < 0.05 was deemed significant.

### Mutation analysis of RS in glioma

Using the TCGA database, we acquired data from 582 cases with somatic mutations and 578 cases with somatic copy number alternations (CNAs) that corresponded with the cases with RNA-seq data. Next, we utilized the R software package “maftool” (23) to screen for various driver genes between the HR and LR patient cohorts. GISTIC2.0 (24) was employed to evaluate CNAs related to RS. Genes with GISTIC value > 1 or < -1 were regarded as amplification or deletion, respectively.

### Statistical analysis

Normally distributed continuous data were evaluated *via* the Shapiro-Wilk test. Kaplan-Meier (KM) was utilized to compare HR and LR patient survival *via* the Log-rank. Stand-alone prognostic markers were identified *via* univariate and LASSO regression models. ROC curves and AUC at the 3‐ and 5‐year follow-ups were computed to examine the predictability of RS using the ‘timeROC’ package. R (version 3.6.3) and its packages were applied for all data analyses (https://www.r-project.org). Two-tailed p-value <0.05 was set as the significance threshold.

## Results

### ScRNA-seq analysis of glioma

**Figure 2:**
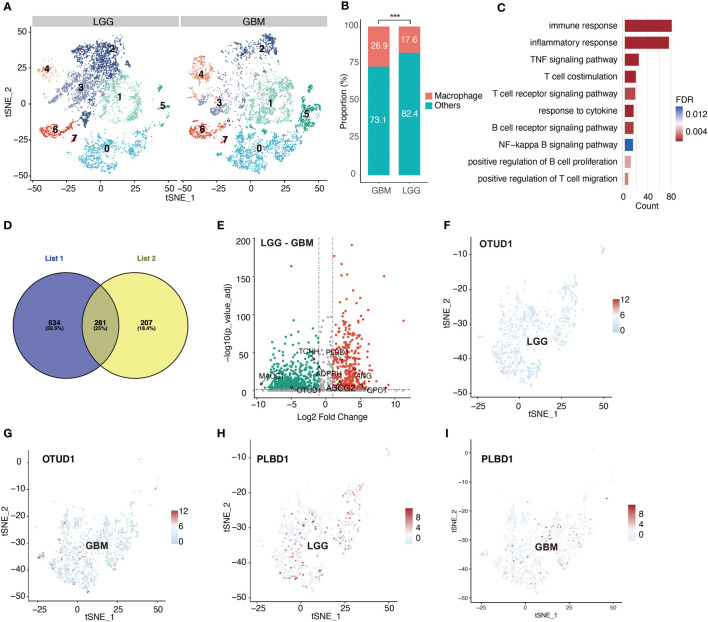
Significant differences emerge in macrophage clusters between LGG and GBM. **(A)** tSNE plot shows cell clusters of LGG and GBM. **(B)** Difference in the proportion of macrophage between LGG and GBM. **(C)** Difference in macrophage biological process between LGG and GBM based on gene enrichment analysis. **(D)** Venn diagram shows macrophage-related genes which are also differentially expressed between LGG and GBM. List1 shows the number of macrophage-related genes. List2 shows the number of differentially expressed genes in macrophage between LGG and GBM. **(E)** Volcano plot shows differentially expressed macrophage-related genes between LGG and GBM. **(F–I)** tSNE plots show the expression of the identified differentially expressed macrophage-related genes. ***P < 0.001.

Following the aforementioned workflow, we retained 15253 (94.9%) high-quality cells, with a median of 4698 RNA features detected within an individual cell from 26 glioma cases. A total of 7365 and 7888 single cells were obtained from LGG and GBM, respectively. Subsequently, all cells were separated into 8 clusters, which were then visualized using t-SNE (**Figure 1B**). According to the CellMarker database, Cluster 0 exhibited markedly elevated levels of CD68, C1QB, CD74, and RNASET2, which were later identified as macrophages (**Figures 1C–F**). We also examined the macrophage quantity and proportion between LGG and GBM (**Figure 2A**). The GBM macrophage proportion was 26.9% (quantity: 2121), whereas the LGG macrophage proportion was only 17.6% (quantity: 1294), which was statistically significant (P < 0.001) (**Figure 2B**). Apart from these differences in macrophage quantity and proportion, there were also differences in macrophage functions (**Figure 2C**) and proliferation (**Figures S3A, B**) between LGG and GBM as well. Gene Ontology analysis revealed that DE-MRGs between LGG and GBM were enriched in immune response, inflammatory response, TNF axis, response to cytokine, and so on.

### Identification and validation of a predictive model

According to a previously described method, we identified 281 DE-MRGs (**Figure 2D**). Next, we explored whether the DE-MRGs were associated with glioma patient prognosis. In total, eight genes were identified (**Figure 2E**). They not only express differentially between LGG and GBM but are also abundant in macrophages (**Figures 2F–I**, **S1A–H, S2A–L**).

**Figure 3:**
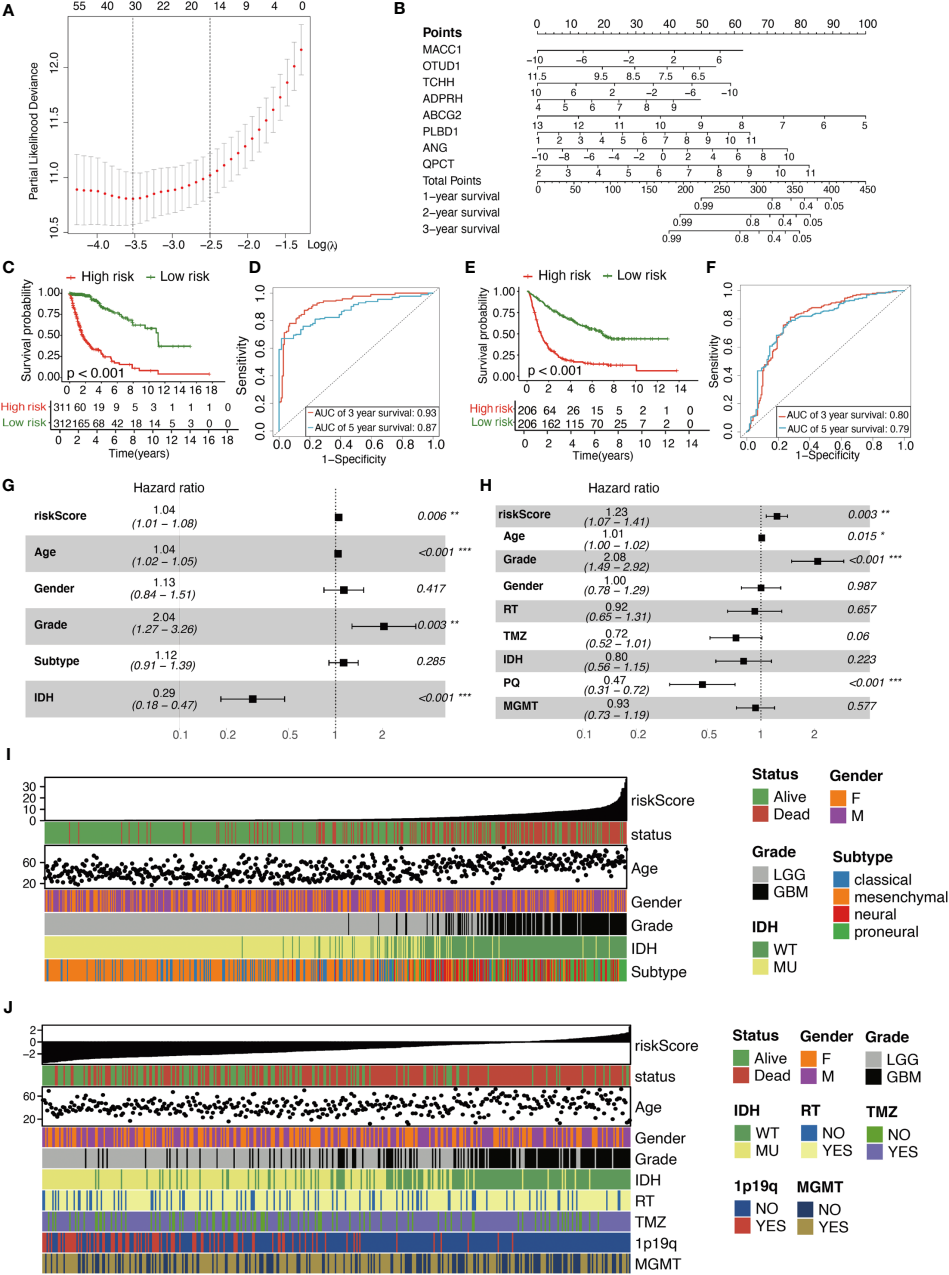
Characterization of the signature predicts prognosis of glioma. **(A)** Texture feature selection using the LASSO regression model, lambda value was chosen (1-SE criteria) according to cross-validation, where optimal l resulted in six non-zero coefficients in the training cohort. **(B)** Nomogram to predict the 1-, 2-, 3-year OS. Kaplan–Meier curve based on the predictive model in TCGA cohort **(C)** and CGGA cohort1 **(E)**. ROC curves of the signature for predicting 3- and 5- year survival of glioma in both TCGA cohort **(D)** and CGGA cohort1 **(F)**. **(G, H)** Multivariable comparison of clinical features and the risk score. Subtype includes classical (reference), mesenchymal, neural, proneural. Risk score is correlated with clinicopathological features and prognosis of glioma in TCGA cohort **(I)** and CGGA cohort1 **(J)**. IDH, isocitrate dehydrogenase; RT, radiotherapy; TMZ, temozolomide; 1p19q, 1p/19q codeletion status; MGMT, methylguanine methyltransferase. *P < 0.05, **P < 0.01, ***P < 0.001.

The detailed calculation process was as follows. First, 218 survival-related DE-MRGs were identified with univariate Cox analysis in TCGA cohort. And then a total of 218 variables were reduced to 16 potential predictors in TCGA cohort (14:1 ratio) by using LASSO analysis (**Figure 3A**). In addition, the features with non- zero coefficients were employed in a multivariate Cox regression model to calculate the risk score of the three cohorts. Subsequently, TCGA cohort, CGGA cohort1, and CGGA cohort2 RSs were computed (**Figure 3B**), as shown below: RS = (0.11 × EXP_MACC1_) + (−0.31 × EXP_OTUD1_) + (−0.09 × EXP_TCHH_) + (0.26 × EXP_ADPRH_) + (-0.40× EXP_ABCG2_) + (0.21 × EXP_PLBD1_) + (0.12 × EXP_ANG_) + (0.29 × EXP_QPCT_). Cases were stratified into two groups, based on the median RS value. Based on the KM of TCGA cohort, RS was a strong prognostic indicator of glioma patient outcome (**Figure 3C**). **Figure 3D** illustrates the AUCs were 0.93 and 0.87 for predicting 3- and 5-year OS, respectively. Similarly, KM analysis also revealed that RS was also markedly correlated with patient OS in the remaining two CGGA cohorts (**Figures 3E** and **S4A**). Moreover, the AUCs were 0.80 and 0.79 for predicting 3- and 5-year OS in CGGA cohort1 and 0.77 and 0.76 for predicting 3- and 5-year OS in CGGA cohort2, respectively (**Figures 3F** and **S4B**). In the meantime, using multivariate analysis, we revealed that RS was a stand-alone indicator of patient OS in the three cohorts (**Figures 3G, H**, **S4C**). The relationships between RS and patient pathological profiles, namely, survival status, WHO grade, IDH status, Subtype, 1p/19q codeletion status, and so on were presented as heatmaps, suggesting that the RS was significantly correlated with these variables in the three cohorts (**Figures 3I, J**, **S4D**). WHO Grade subset analyses confirmed the prognostic value of RS, particularly in WHO II and III grade glioma (**Figures S5A–F**). Besides, IDH status subset demonstrated the stable prognostic value of RS in both IDH -mutated and IDH -wildtype cases (**Figures S5G–L**).

### Role of RS in TME immunity

**Figure 4:**
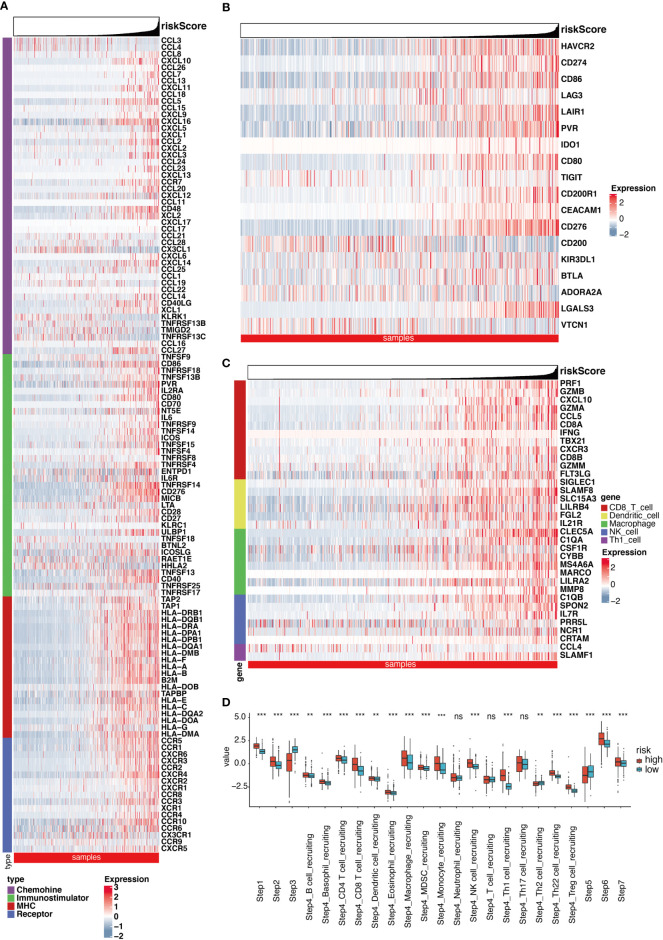
Significant differences exist in immune landscape. **(A)** Differences in the expression of 121 immunomodulators (chemokines, receptors, MHC, and immunostimulators) between high- and low-risk score groups in glioma. **(B)** Differences in the expression of 18 inhibitory immune checkpoints between high- and low-risk score groups in glioma. **(C)** Differences in the effector genes of the tumor-associated immune cells between high- and low-risk score groups in glioma. **(D)** Differences in the various steps of the cancer immunity cycle between high- and low-risk score groups. **P < 0.01, ***P < 0.001, ns non-significant.

After removing the unexpressed IMs, we obtained 121, 108, and 107 related genes from TCGA cohort, CGGA cohort1 and CGGA cohort2, respectively. These included MHC molecules, chemokines, immunostimulators, and receptors. A majority of the IMs were elevated in the enhanced RS cohorts (**Figures 4A**, **S6A, S7A**). Furthermore, the elevated IMs were strongly associated with antigen-presenting activity and TIICs recruitment. Consistently, we demonstrated that the RS was intricately linked to most ICIs and TIICs effector genes in the three cohorts (**Figures 4B, C**, **S6B, C**, **S7B, C**). The CIC served an essential function in the TME. Relative to the low RS cohort, most genes were augmented (**Figure 4D**). Compared with the LR group, as shown in **Figure 4D**, most of the steps were upregulated. Nevertheless, Step 3, Step4_Th2 cell_recruiting, and Step 5 were diminished. Similar results were obtained from the two CGGA cohorts (**Figures S6D**, **S7D**). Besides, the positive correlation between RS and the infiltration level of macrophage in TME (including two subgroups, macrophages M1 and macrophages M2) was further investigated using three different algorithms in the three cohorts (**Figures S8A–R**). Meanwhile, we further explored the relationship between RS and classical chemokines and surface markers of both M1-macrophages (IL12A, IL-12B, IL-23A, IL-23R, TNF) and M2-macrophages (IL-10, IL-4, IL-13, TGF-beta 1, TGF-beta 2, TGF-beta 3). After the removal of the unexpressed markers, as the result, RS was positively correlated with the most of chemokines related to macrophages (**Figures S9A–H**). Comparable results were also achieved using the remaining two CGGA cohorts (**Figures S10A–E**, **S11A–E**). These analyses revealed that RS played a critical role in facilitating immunological activities.

### Role of RS in biological processes

We conducted enrichment analysis to further clarify biological processes related to RS. The 1306 genes in TCGA cohort, 898 genes in CGGA cohort1, and 644 genes in CGGA cohort2 were analyzed by enrichment analysis, and they were strongly associated with RS by Pearson correlation analysis (Pearson |r| > 0.5, P < 0.05). As illustrated in **Figure S12A**, enrichment analysis indicated that GO or KEGG is mainly enriched in inflammatory response, cell migration, cell-cell adhesion, tight junction, and so on. In the other two cohorts, similar outcomes were obtained (**Figures S12B, C**).

### Correlation between RS and genomic alterations

**Figure 5:**
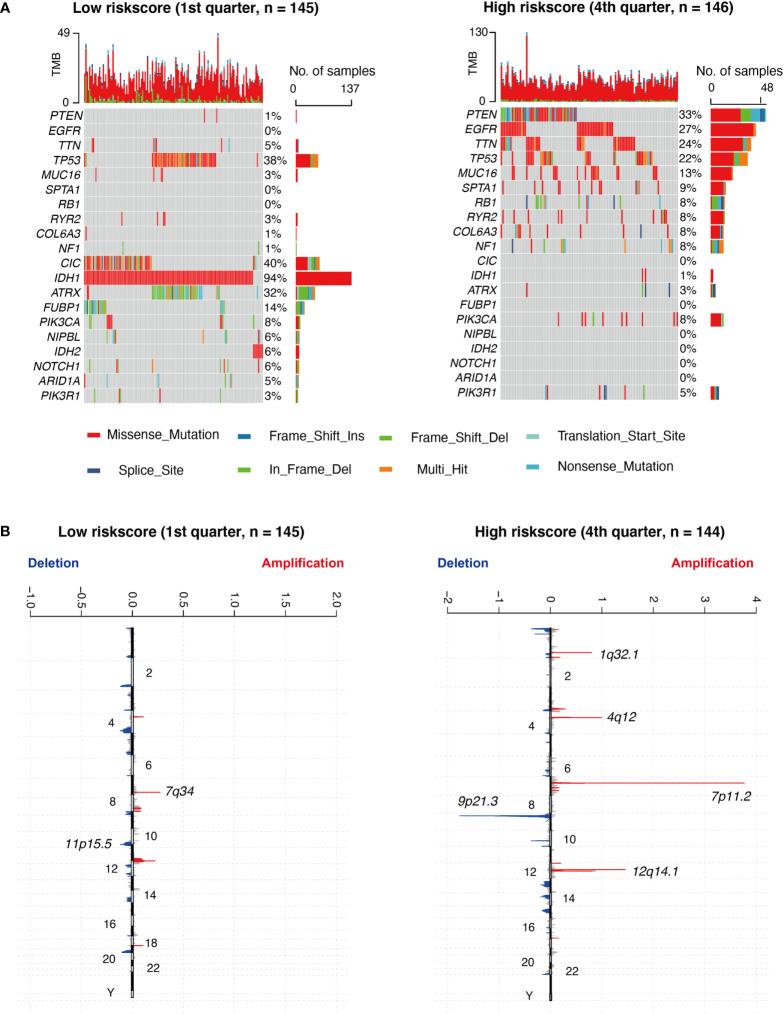
Different genomic profiles are associated with risk score. **(A)** Differential somatic mutations were detected by comparing glioma with low- and high- risk score groups. **(B)** A different CNAs profile could be observed between low- and high- risk score groups. Chromosomal locations of peaks of significantly focal amplification (red) and deletions (blue) were presented.

Based on a high to low ranking of the RS, we stratified cases into four categories. First, we compared the gene mutation frequencies in the 1st quarter (lower) with that of the 4th quarter (higher) RS cohort. Based on our analysis, IDH1, ATRX, FUBP1, TP53, CIC, NIPBL, IDH2, NOTCH1, and ARID1A mutations were more frequent in the lower RS cohort. In contrast, PTEN, EGFR, TTN, MUC16, SPTA1, RB1, RYR2, COL6A3, NF1, and PIK3R1 mutations were more prevalent in the higher RS cohort (**Figure 5A**). In terms of the CNAs analysis, cases with elevated RS, focal amplification peaks, well-characterized driver oncogenes like PIK3C2B (1q32.1), PDGFRA (4q12), EGFR (7p11.2), and CDK4 (12q14.1), were accompanied by a 9p21.3 (CDKN2A and CDKN2B) focal deletion peak. In the meantime, obvious amplifications revealed peaks in 7q34, whereas the frequently deleted genomic regions were 11p15.5 in the lower RS cohort. However, the corresponding G scores did not reach the threshold that defined abnormal CNA events (**Figure 5B**).

## Discussion

Glioma, particularly GBM, is a widespread brain tumor that is hazardous to health and has high mortality owing to its malignant progression and worse outcome. TME and glioma heterogeneity are rather complicated (4, 25), and they are still unclear at present. Generally, relative to bulk sequencing, single-cell RNA sequencing technologies facilitate gene expression exploration at the single-cell level. This provides unparalleled insight into the cellular heterogeneity of biological pathways. Previous studies primarily examined DEGs in the TME or screened for biomarkers in bulk sequencing for the construction of prediction models. Herein, we identified DE-MRGs, and generated a DE-MRG-based prognostic model to accurately predict patient OS. We also explored the correlations between the prognostic model and various clinical features. With emerging research, the role of macrophages in glioma is gradually expanding.

TAMs strongly modulate neoplasia, metastasis, immune escape, and tumor angiogenesis (26, 27). TAMs between LGG and GBM are also dramatically different. Specifically, the proportion of proliferating TAMs (G2M and S phage) is higher in LGG (28), while the proportion of TAMs is higher in GBM (29), which is in agreement with our study. Apart from the differences in these aspects, functional differences also exist between these two groups. Multiple factors mediate TAM recruitment, activation, and polarization. These include chemokines, complement receptor ligands, and neuro- transmitters, such as, CCL2 (30) and SDF-1 (31). These factors have marked differential expression between LGG and GBM.

In our study, we identified eight macrophage-specific genes, which were MACC1, OTUD1, TCHH, ADPRH, ABCG2, PLBD1, ANG, and QPCT. As previously published, MACC1 (32) and ADPRH (33) ABCG2 (34), and ANG (35) correlate with glioma cell proliferation, invasion, immune infiltration, drug efficacy, and worse prognosis in glioma patients. Hence, it is not surprising that this model showed superior performance in predicting worse patient outcomes. Interestingly, not much is known about the functions of OTUD1, TCHH, ADPRH, PLBD1, and QPCT in glioma, which need further research in future. Additionally, a majority of these findings were made by investigating the bulk sequencing data, and there was no specific mention of which cell type these genes were expressed in. In the current study, multi-omics approaches were applied to construct a prognostic model to estimate glioma patient OS. We also noted that these genes were specifically and highly expressed genes in macrophages, thus laying the foundations for future treatment in precision oncology medicine. Interestingly, RS is not only significantly associated with immune checkpoint markers, inflammatory factors, and immune steps, but also with the infiltration level of macrophage and chemokines related to macrophages. It shows RS is a composite indicator. Besides, except for the immune response, gene enrichment analysis shows RS is related to cell migration, regulation of cell shape, and cell adhesion, which was a clearly defined relationship with tumor invasion and poor prognosis in glioma (36, 37). These results might show the reason why RS exhibits accurately predictive performance for the survival of glioma patients.

In addition, we assessed genetic alterations that occurred in patients with low versus high RS. Upon close observation of somatic mutation events, CNAs were closely correlated with RS, indicating an unstable genomic status in high RS patients. Generally, genomic alterations occur in glioma cells, and they are correlated with drug resistance, poor prognosis, and tumor aggressiveness. However, the risk model based on DE-MRGs was still associated with genomic alterations. Genomic alterations and heterogeneity may have substantial roles in editing the glioma TME. Elevated RS induces an intensive immune phenotype that further aggravates genomic instability (38), thus creating a positive feedback that exacerbates poor prognosis and treatment resistance (39). However, the question remains whether the genomic alteration observed between LR and HR patients is a consequence or a cause of the differences in macrophages between glioma patients. More research is warranted to elucidate this unanswered question.

## Conclusion

This study confirmed that the proportion and function of macrophages in glioma TME are significantly different. Moreover, we developed a DE-MRG-based prognostic model which accurately predicted patient prognosis, and may, therefore, be applicable to the development of therapeutic targets.

## Data availability statement

Data are available in a public, open access repository. The datasets analyzed during the current study are available in the Gene Expression Omnibus (GSE89567: https://www.ncbi.nlm.nih.gov/geo/query/acc.cgi?acc=GSE89567; GSE84465: https://www.ncbi.nlm.nih.gov/geo/query/acc.cgi?acc=GSE84465) and The Cancer Genome Atlas (TCGA) (https://cancergenome.nih.gov/) and the Chinese Glioma Genome Atlas (http://www.cgga.org.cn/).

## Ethics statement

Written informed consent was obtained from the individual(s), and minor(s)’ legal guardian/next of kin, for the publication of any potentially identifiable images or data included in this article.

## Author contributions

YW, XG, and WJ conceptualized and designed the study. YW, XG, YL, and CKZ acquired the data. YW, YL, and CBZ analyzed and interpreted the data. YW wrote and reviewed the manuscript. WJ and XG supervised the study. All authors contributed to the article and approved the submitted version.

## Funding

This project was supported by the National Natural Science Foundation of China (No. 82071996, No. 81802483 and No. 82003075), the Beijing Municipal Health Commission of China (No. PXM2019_026280_000002), the Capital’s Funds for Health Improvement and Research (No. 2018-1-1071), and Beijing Hospitals Authority Youth Program (No. QML20190507 and No.QML20210502).

## Conflict of interest

The authors declare that the research was conducted in the absence of any commercial or financial relationships that could be construed as a potential conflict of interest.

## Publisher’s note

All claims expressed in this article are solely those of the authors and do not necessarily represent those of their affiliated organizations, or those of the publisher, the editors and the reviewers. Any product that may be evaluated in this article, or claim that may be made by its manufacturer, is not guaranteed or endorsed by the publisher.
