# Supplementary material for: Differences of macrophages in the tumor microenvironment as an underlying key factor in glioma patients
Source: Front Immunol. 2022 Oct 31;13:1028937. doi: 10.3389/fimmu.2022.1028937 (PMC9659848; doi:10.3389/fimmu.2022.1028937)
Supplement: Supplementary file 1 [file DataSheet_1.docx]

Supplementary Material

# Supplementary Figures and Tables

## Supplementary Figure


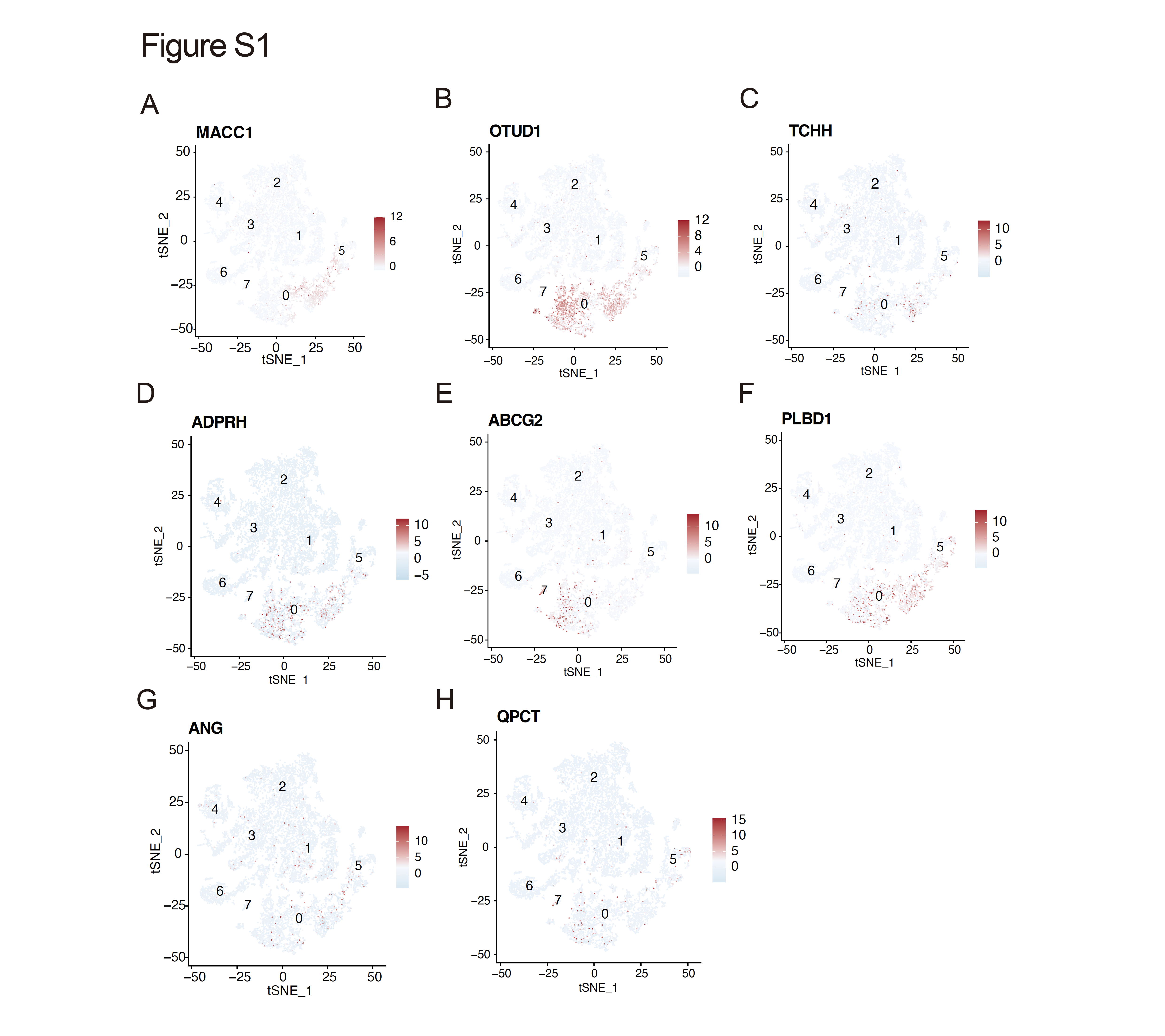


**Supplementary Figure S1.** (A-H) tSNE plots show the expression of identified eight genes between cluster0 and others. Cluster0 is identified as macrophage group.


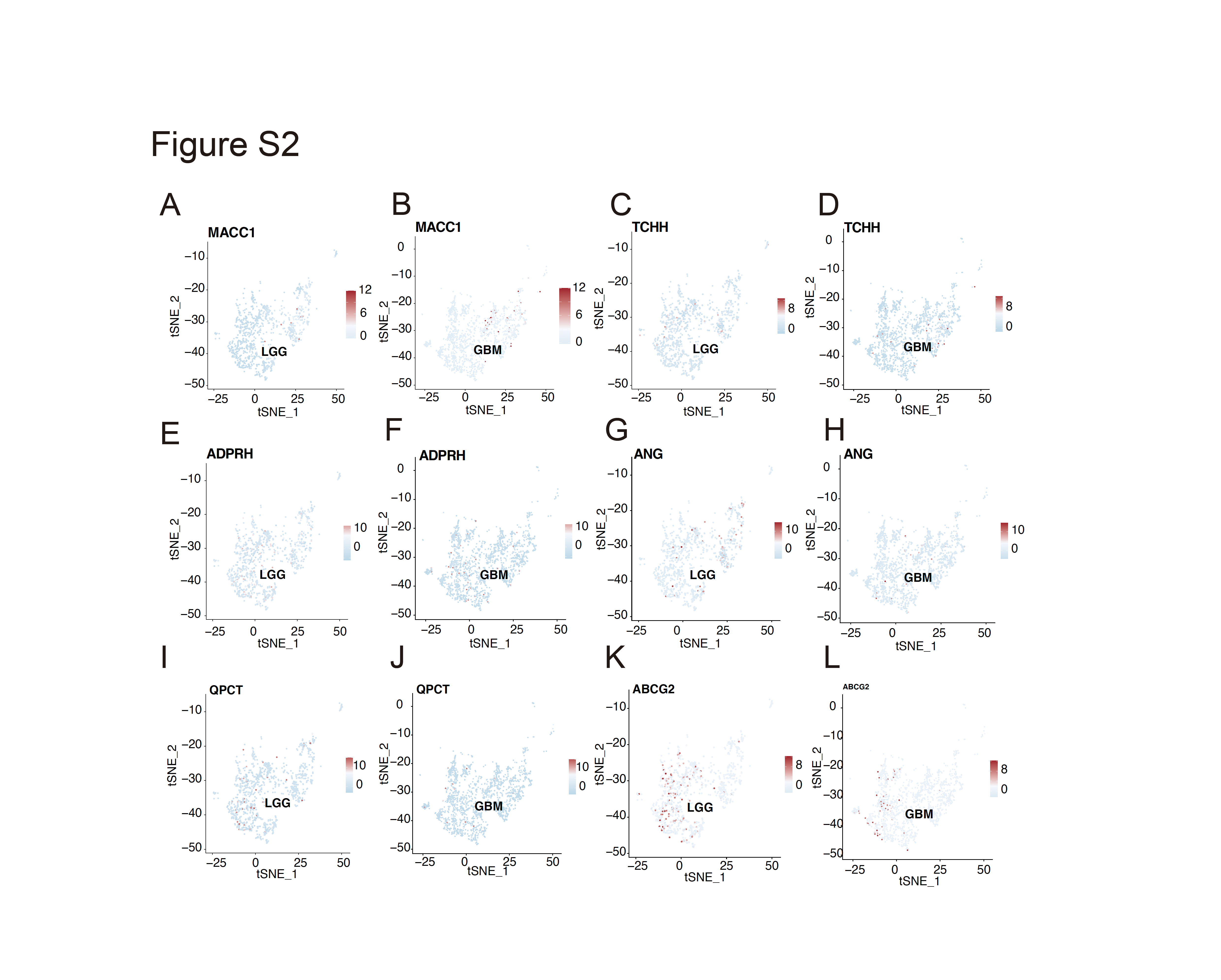


**Supplementary Figure S2.** (A-L) tSNE plots show identified differentially expressed macrophage-related genes in cluster0 subgroup between LGG and GBM. Cluster0 was identified as macrophage group. LGG: lower grade glioma; GBM: glioblastoma.


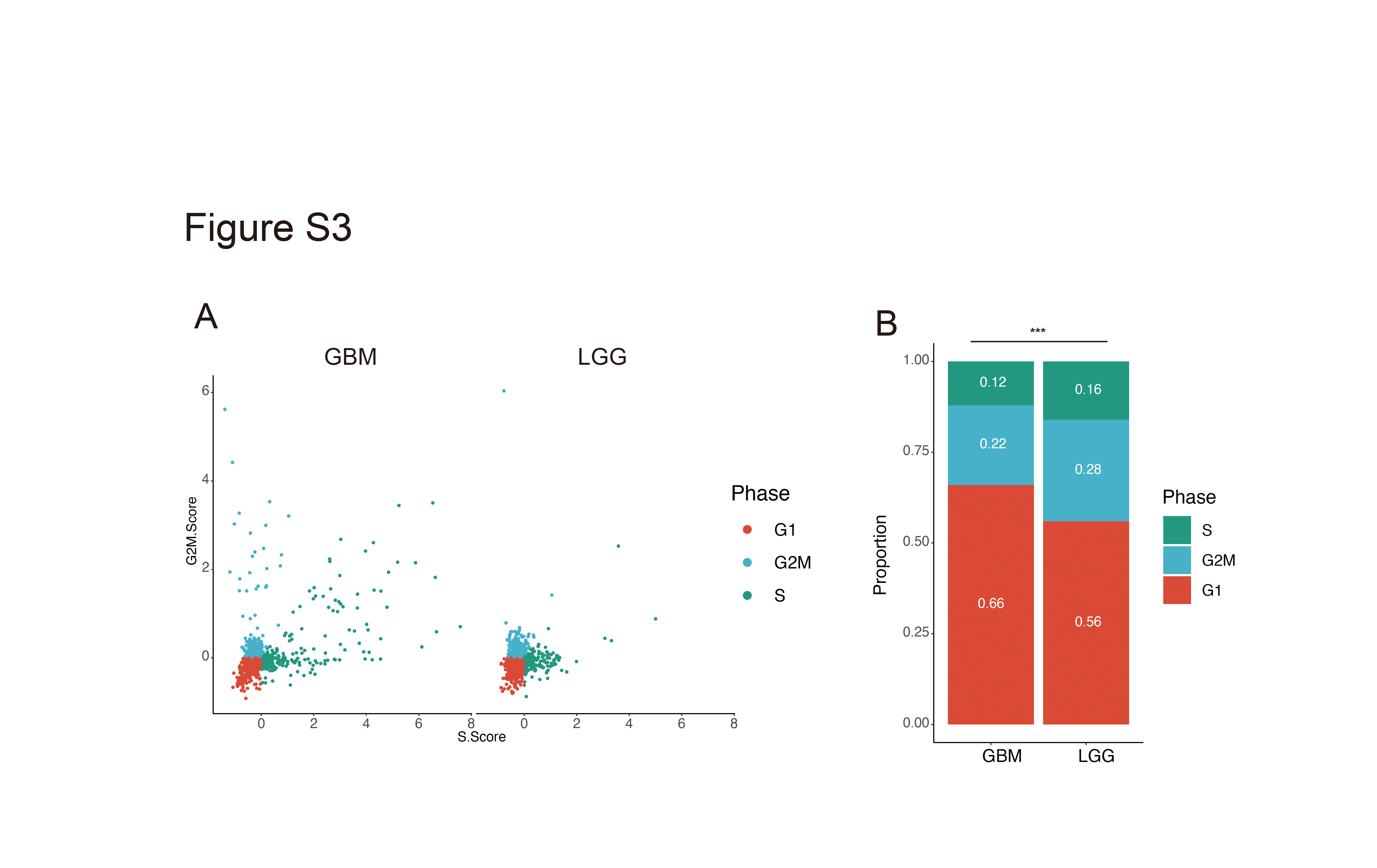


**Supplementary Figure S3.** Scatter plot and column plot show the different proportion of cell cycle phase of macrophage between LGG and GBM. The statistical difference was apparent. LGG: lower grade glioma; GBM: glioblastoma. ***P < 0.001.

**Supplementary Figure S4.** The prognosis values of risk score in CGGA cohort2. (A) Kaplan–Meier curve based on the predictive model indicated that high risk group conferred poor prognosis. (B) ROC curves of the signature for predicting 3- and 5- year survival of glioma in CGGA cohort2. (C) Multivariable comparison of clinical features and the risk score in CGGA cohort2. (D) Risk score is correlated with clinicopathological features and prognosis of glioma in CGGA cohort2. IDH = isocitrate dehydrogenase; 1p19q = 1p/19q codeletion status; MGMT = methylguanine methyltransferase.

**Supplementary Figure S5.** The prognosis values of risk score in subgroup analysis. Kaplan–Meier curve of WHO grade subgroup based on risk score in TCGA cohort (A-B), CGGA cohort1 (C-D), and CGGA cohort2 (E-F). Kaplan–Meier curve of IDH status subgroup based on risk score in TCGA cohort (G-H), CGGA cohort1 (I-J), and CGGA cohort2 (K-L). IDH: isocitrate dehydrogenase


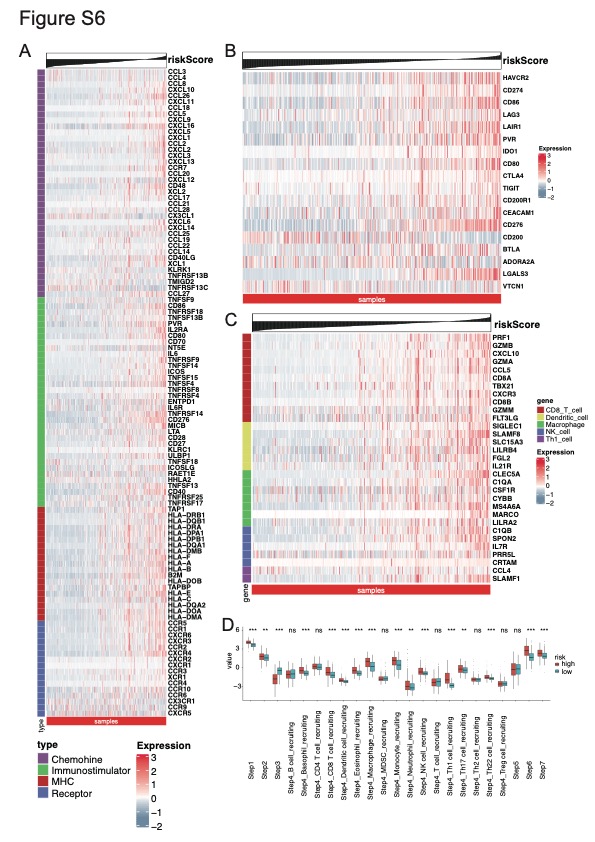


**Supplementary Figure S6.** Significant differences exist in immune landscape in the CGGA cohort1. (A) Differences in the expression of 108 immunomodulators (chemokines, receptors, MHC, and immunostimulators) between high- and low-risk score groups in glioma. (B) Differences in the expression of 18 inhibitory immune checkpoints between high- and low-risk score groups in glioma. (C) Differences in the effector genes of the tumor-associated immune cells between high- and low-risk score groups in glioma. (D) Differences in the various steps of the cancer immunity cycle between high- and low-risk score groups. **P < 0.01, ***P < 0.001, ns non-significant


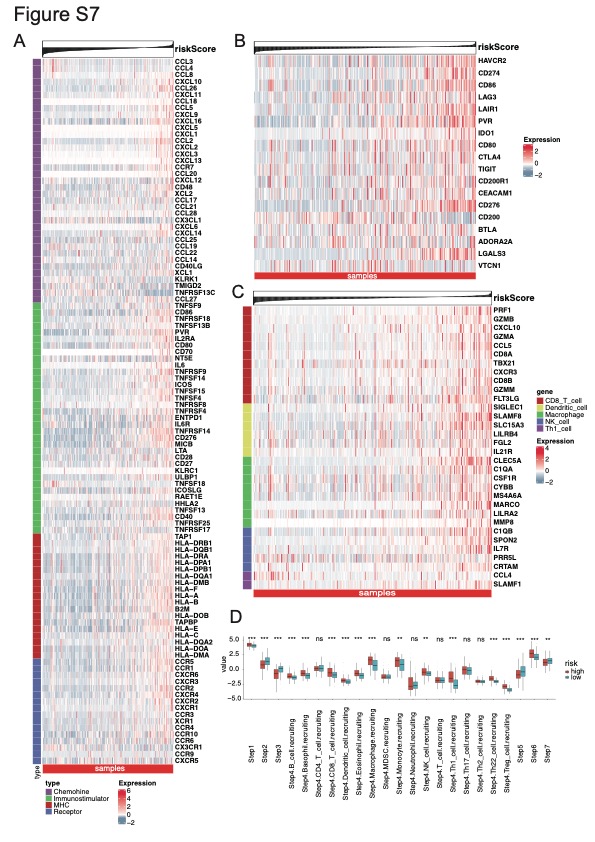


**Supplementary Figure S7.** Significant differences exist in immune landscape in the CGGA cohort2. (A) Differences in the expression of 107 immunomodulators (chemokines, receptors, MHC, and immunostimulators) between high- and low-risk score groups in glioma. (B) Differences in the expression of 18 inhibitory immune checkpoints between high- and low-risk score groups in glioma. (C) Differences in the effector genes of the tumor-associated immune cells between high- and low-risk score groups in glioma. (D) Differences in the various steps of the cancer immunity cycle between high- and low-risk score groups. **P < 0.01, ***P < 0.001, ns non-significant


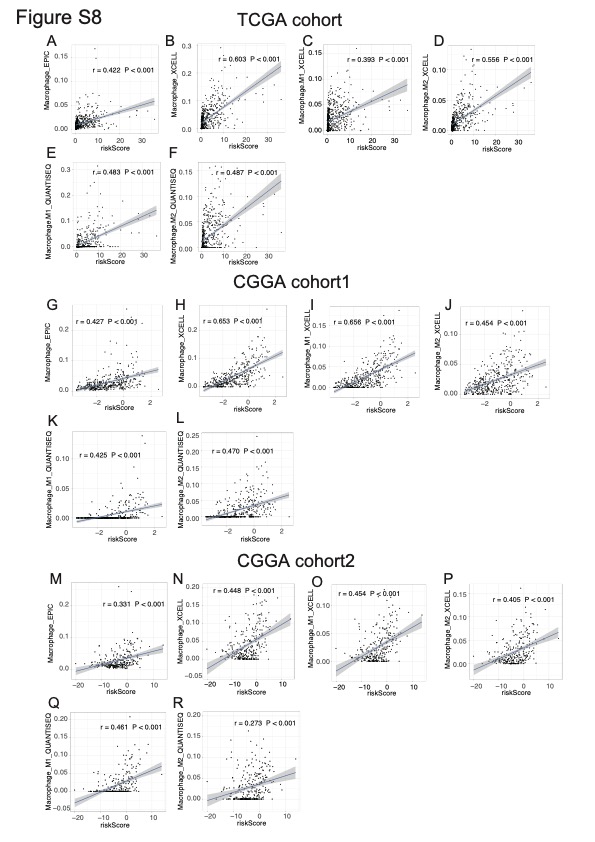


Correlation of risk score and the infiltration levels of macrophage and subclusters (M1 and M2) in TCGA cohort (A-F), CGGA cohort1 (G-L), and CGGA cohort2 (M-R). EPIC, XCELL, QUANTISEQ are three independent algorithms to infer the infiltration levels of macrophage using bulk RNA-seq data.


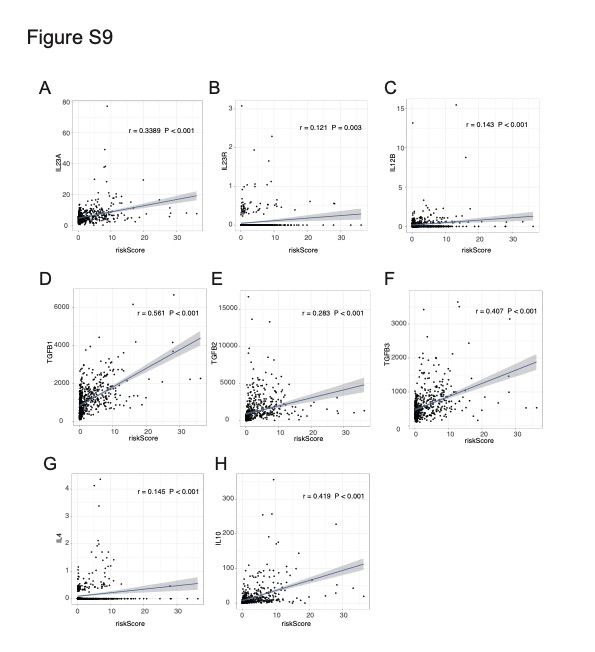
Correlation of risk score and classical chemokines and surface markers of macrophage in TCGA cohort. (A-C) IL-23A, IL-23R, and IL-12B are M1 macrophage chemokines. (D-H) TGFB1, TGFB2, TGFB3 IL4, and IL10 are M2 macrophage chemokines.


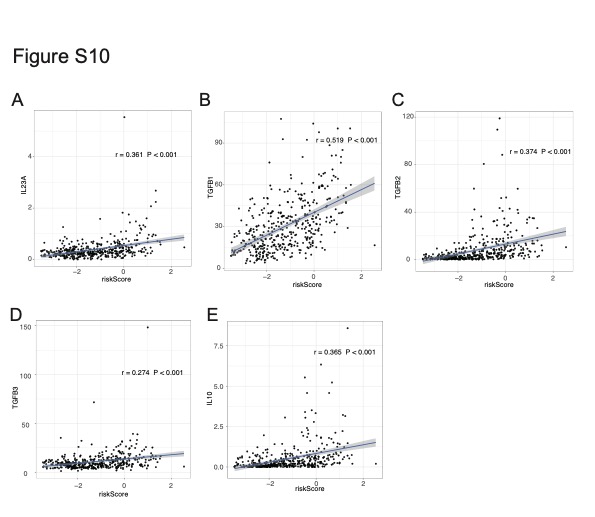


Correlation of risk score and classical chemokines and surface markers of macrophage in CGGA cohort1. (A) IL-23A is M1 macrophage chemokine. (B-E) TGFB1, TGFB2, TGFB3, and IL10 are M2 macrophage chemokines


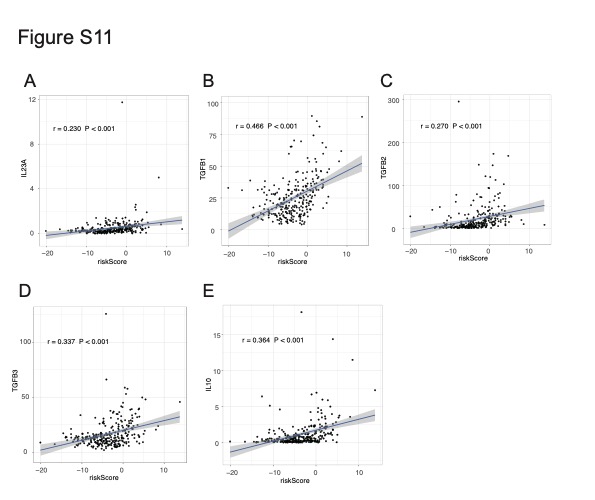


Correlation of risk score and classical chemokines and surface markers of macrophage in CGGA cohort2. (A) IL-23A is M1 macrophage chemokine. (B-E) TGFB1, TGFB2, TGFB3, and IL10 are M2 macrophage chemokines

Enrichment analysis including GO and KEGG among genes strongly related to risk score in TCGA cohort (A), CGGA cohort1 (B), and CGGA cohort2 (C). Bar length shows gene counts enriched in the enrichment of pathway. Color depth indicates the p value from low (purple) to high level (red). The p values of all presented hits are less than 0.05

## Supplementary Table

| **Supplementary table 1. Patient characteristics from three cohorts.** | | | |
| --- | --- | --- | --- |
| **Clinical characteristics** | **TCGA cohort** | **CGGA cohort1** | **CGGA cohort2** |
| **Age at diagnosis (year)** |  |  |  |
| ≤45 | 300 (48.2%) | 247 (60.0%) | 164 (60.1%) |
| >45 | 323 (51.8%) | 165 (40.0%) | 109 (39.9%) |
| **Gender** |  |  |  |
| Female | 264 (42.4%) | 181 (43.9%) | 105 (38.5%) |
| Male | 359(57.6%) | 231 (36.1%) | 168 (61.5%) |
| **Grade** |  |  |  |
| LGG | 463 (74.3%) | 257 (62.4%) | 146 (53.5%) |
| GBM | 160 (25.7%) | 155 (37.6%) | 127 (46.5%) |
| **Subtype** |  |  |  |
| classical | 97 (15.7%) | NA | NA |
| mesenchymal | 102 (16.4%) | NA | NA |
| neural | 121 (19.4%) | NA | NA |
| proneural | 303 (48.5%) | NA | NA |
| **IDH mutation** |  |  |  |
| No | 237 (38.0%) | 185 (44.9%) | 130 (47.6%) |
| Yes | 386 (62.0%) | 227 (55.1%) | 143 (52.4%) |
| **1p19q codeletion** |  |  |  |
| No | NA | 325 (78.9%) | 219 (80.2%) |
| Yes | NA | 87 (21.1%) | 54 (19.8%) |
| **MGMT** |  |  |  |
| Unmethylated | NA | 168 (40.8%) | 131 (48.0%) |
| Methylated | NA | 244 (59.2%) | 142 (52.0%) |
| **Radiation therapy** |  |  |  |
| No | NA | 84 (20.4%) | 57 (20.9%) |
| Yes | NA | 328 (79.6) | 216 (79.1%) |
| **TMZ** |  |  |  |
| No | NA | 88 (21.4%) | 96 (35.2%) |
| Yes | NA | 324 (78.6%) | 177 (64.8%) |
| NA: unavailable |  |  |  |
